# The influence of dietary peptide inhibitors of angiotensin-converting enzyme on the hypotensive effects of enalapril

**DOI:** 10.1186/s40780-015-0018-3

**Published:** 2015-06-03

**Authors:** Machiko Watanabe, Junichi Kurihara, Shigeto Suzuki, Kazuki Nagashima, Hiroyuki Hosono, Fumio Itagaki

**Affiliations:** Laboratory of Clinical Pharmaceutics, Faculty of Pharma Sciences, Teikyo University, Kaga 2-11-1, Itabashi-ku, Tokyo, 173-8605 Japan; Laboratory of Medical Pharmacology, Faculty of Pharma Sciences, Teikyo University, Kaga 2-11-1, Itabashi-ku, Tokyo, 173-8605 Japan

**Keywords:** Hypertension, Drug interaction, Fermented milk, Enalapril, Peptide, Angiotensin I-converting enzyme inhibitor, Concomitant administration, Foods for specified health uses

## Abstract

**Background:**

Enalapril is an antihypertensive medicine that inhibits angiotensin I-converting enzyme (ACE). The present study investigated interactions between enalapril and a fermented milk product (FMP) containing the ACE-inhibitory peptides, Val-Pro-Pro (VPP) and Ile-Pro-Pro (IPP).

**Methods:**

Single-dose and long-term (6-week) *in vivo* studies were used to investigate the effects of enalapril and FMP on blood pressure in spontaneously hypertensive rats.

**Results:**

Single-dose oral administration of concomitant enalapril and FMP (VPP, IPP: 3.5 mg/kg) produced a lower antihypertensive effect than enalapril monotherapy. However, this effect was not observed in animals administered a lower dose of FMP (VPP, IPP: 1.75 mg/kg) along with enalapril. In rats administered enalapril concomitantly with a fish protein product (FPP) containing a different ACE inhibitory peptide (Leu-Lys-Pro-Asn-Met), significant attenuation of the antihypertensive effect was also observed 1 and 2 h after administration, as compared to enalapril monotherapy. During a 6-week oral administration study, the enalapril monotherapy group showed significant antihypertensive effects compared to those observed in the controls on day 28. Oral administration of enalapril and FMP, with a 1-h interval between doses, resulted in significant antihypertensive effects on day 35, indicating a delayed onset in comparison to enalapril monotherapy. In rats receiving enalapril monotherapy for 28 days, followed by 14 days of concomitant FMP, significant antihypertensive effects were observed after day 35, and these did not differ significantly from the effects observed during enalapril monotherapy.

**Conclusions:**

The present findings suggested that long-term concomitant intake of FMP and enalapril could influence the antihypertensive effects of this drug.

## Background

Foods for Specified Health Uses (FOSHU) have been approved by the Japanese Ministry of Health, Labour and Welfare after demonstrating tangible benefits. FOSHU may contain biologically active ingredients with a range of activities, such as decreasing blood pressure or cholesterol levels. These provide prophylaxis for lifestyle-related diseases, such as hypertension, diabetes, obesity, osteoporosis, and cardiovascular disease [1]. At present, many FOSHU such as tea, beverages, gum, etc. are available over-the-counter and have been widely adopted in everyday life.

Hypertension can be treated using lifestyle measures, such as increasing physical activity, maintaining a normal body weight, and adopting a healthy diet. In addition, borderline hypertensive subjects can ingest FOSHU containing peptide inhibitors of angiotensin I-converting enzyme (ACE). Several randomized trials and meta-analyses have shown that some peptides derived from milk proteins, such as Val-Pro-Pro (VPP) and Ile-Pro-Pro (IPP), decrease systolic blood pressure [2–4]. VPP and IPP, first isolated from milk fermented with Lactobacillus helveticus, show ACE-inhibitory activity and their antihypertensive effects have been studied extensively in spontaneously hypertensive rats (SHR) [5, 6], and in humans [7, 8]. At present, a fermented milk product (FMP) containing these peptides is commercially available in Japan (trade name, Ameel S®) and this FOSHU is readily available to people, irrespective of whether they are taking other antihypertensive medications.

There are few published studies on the effects of combined intake of FMP and a prescribed ACE inhibitor. The present study therefore investigated the hypotensive effects of concomitant use of FMP and an ACE inhibitor, enalapril. Because antihypertensive drugs are commonly administered continuously over a long period, this study investigated long-term administration of FMP and enalapril. An initial investigation of concomitant oral administration of enalapril and either FMP or a commercially available fish protein product (FPP; trade name, Peptide Straight®) containing a bioactive peptide (Leu-Lys-Pro-Asn-Met; LKPNM) was carried out [9]. We then conducted a long-term oral administration study to model a situation where patients took concomitant FOSHU and enalapril, and the other scenario where patients already taking oral antihypertensive medication started to take FOSHU. SHR therefore received daily oral concomitant enalapril and FMP for 6 weeks, or enalapril monotherapy for 28 days, followed by concomitant FMP for a further 14 days.

## Methods

### Animals

This study was performed in accordance with the guidelines for animal experimentation of Teikyo University. Male SHR rats were purchased from the Sankyo Laboratory Service Corporation (Tokyo, Japan) and then underwent a 4-week preliminary observation at 22-24 °C, 55 % humidity, with 12 h of lighting (0800–2000) and food and water *ad libitum*. Thirteen-week-old rats (body weight: 250–290 g) were used for the single administration study and 15-week-old rats (body weight: 235–270 g) were used for the long-term administration study.

### Sample preparation

One Ameel S Handy Tab® (CALPIS Co. Ltd., Tokyo, Japan), labeled as containing 1.7 mg of VPP and IPP in total, was crushed and dissolved in distilled water to give a total volume of either 2.9 mL (for dosing at 3.5 mg/kg [5]) or 5.8 mL (for dosing at 1.75 mg/kg). In the enalapril monotherapy group, 0.5 mg/mL enalapril maleate (Wako Junyaku Inc., Tokyo, Japan) solution was prepared using distilled water. For concomitant enalapril and FMP administration, 1 Ameel S Handy Tab® was crushed and added to a 1.0 mg/mL enalapril solution to give a total volume of 2.9 mL. In the FPP monotherapy group, one bag containing 1.9 g of Peptide Straight® (Japan Supplement Foods Co. Ltd., Osaka City, Japan; 4.75 mg LKPNM) was crushed and dissolved in distilled water to obtain a total volume of 3.5 mL. In the concomitant FPP and enalapril group, 1.9 g Peptide Straight® was crushed and added to a 0.4 mg/mL enalapril solution to give a total volume of 3.5 mL. The control group was orally administered 6 mL/kg distilled water.

### Test product administration

Three types of single administration studies were conducted using the following 4 groups (*n* = 4 rats per group): Study 1, (I) control (distilled water), (II) enalapril (3 mg/kg) monotherapy, (III) FMP (VPP, IPP: 3.5 mg/kg), and (IV) concomitant enalapril (3 mg/kg) and FMP (VPP, IPP: 3.5 mg/kg); Study 2, (I) control (distilled water), (II) enalapril (3 mg/kg) monotherapy, (III) FMP (VPP, IPP: 1.75 mg/kg), and (IV) concomitant enalapril (3 mg/kg) and FMP (VPP, IPP: 1.75 mg/kg); and Study 3, (I) control (distilled water), (II) enalapril (3 mg/kg) monotherapy; (III) FPP (LKPNM; 10 mg/kg LKPNM [9].), and (IV) concomitant enalapril (3 mg/kg) and FPP (LKPNM; 10 mg/kg LKPNM). These doses of FMP and enalapril were based on the human doses of 5–10 mg enalapril daily and 3.4 mg VPP/IPP in FMP. The test products were administered orally using a nasogastric tube (KN-349, Natsume, Tokyo, Japan) at the scheduled time (1000–1145). In the long-term administration study, rats received each preparation separately, with an interval of 1 h between the first and second administration. This study was conducted using the following groups and dosing schedules (*n* = 5 rats per group): (I) control (distilled water, followed by distilled water); (II) enalapril monotherapy (distilled water, followed by 3 mg/kg enalapril); (III) concomitant enalapril and FMP (VPP, IPP: 3.5 mg/kg), followed by 3 mg/kg enalapril); and (IV) delayed combination (distilled water, followed by 3 mg/kg enalapril for 28 days, switching to FMP [VPP, IPP: 3.5 mg/kg], followed by 3 mg/kg enalapril for a further 14 days). During the 6-week study period, administration took place daily at scheduled times (first administration: 1130–1200; second administration: 1230–1300).

### Blood pressure measurement

Systolic blood pressure was measured by the tail cuff method using the Non-Preheating, Non-Invasive Blood Pressure Monitor for Mice and Rats (MK-2000, Muromachi Kikai, Tokyo, Japan). Blood pressure measurements were performed 5–10 times consecutively when the rat was at rest. During the single administration study, blood pressure was measured immediately before administration and at 1, 2, 4, and 6 h after administration. During the long-term administration study, systolic blood pressure was measured immediately before the start of administration and prior to oral dosing on days 3, 7, 10, 14, 17, 21, 24, 28, 31, 35, 38, and 42 (after day 42, no test product was administered). This study schedule was based on a previous report [10].

A change in the hypertensive state was calculated as a percentage of systolic blood pressure measured immediately before the start of administration.

### Statistical analyses

The mean blood pressure value was used for analysis. Values were expressed as the mean ± standard error of the mean (SE). After one-way analysis of variance, the Bonferroni/Dunn or Scheffe multiple comparison test was performed.

## Results and discussion

### Single administration study

**Fig. 1:**
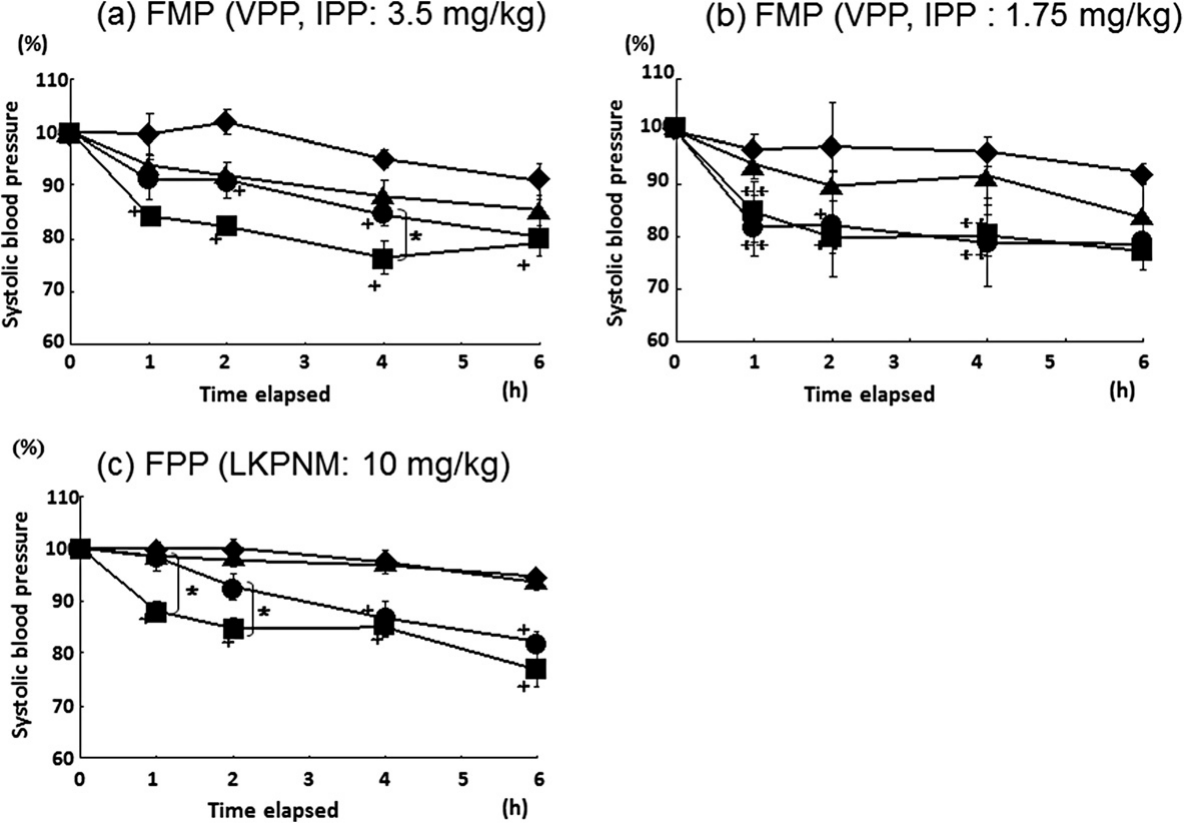
Systolic blood pressure changes in SHR after single oral administration. Systolic blood pressure changes in SHR after single oral administration of **(a)** enalapril (3.0 mg/kg, ■), FMP (VPP, IPP: 3.5 mg/kg, ▲), or concomitant enalapril (3.0 mg/kg) and FMP (VPP, IPP: 3.5 mg/kg, ●); **(b)** enalapril (3.0 mg/kg, ■), FMP (VPP, IPP: 1.75 mg/kg, ▲), or concomitant enalapril (3.0 mg/kg) and FMP (VPP, IPP: 1.75 mg/kg, ●); **(c)** enalapril (3.0 mg/kg, ■), FPP (LKPNM: 10 mg/kg, ▲), or concomitant enalapril (3.0 mg/kg) and FPP (LKPNM: 10 mg/kg, ●). The control group (◆) received distilled water. Values are presented as the mean ± SE (*n* = 4). *p < 0.05 vs enalapril monotherapy group; ^✢^p < 0.05; ^✢✢^p < 0.01 vs the control group at the same time point (Bonferroni/Dunn, Scheffe’s F test)

In the initial single oral dose study, blood pressure was measured after administration of a mixture of enalapril and FMP, to evaluate the optimal method of administration for the subsequent long-term study. As a result, compared to the control group, the enalapril monotherapy group showed significant antihypertensive effects from 1 to 6 h after administration. No significant antihypertensive effects was observed in the concomitant enalapril and FMP (VPP, IPP: 3.5 mg/kg) group 1 h after administration, although significant antihypertensive effects were observed at 2 and 4 h (p < 0.05 each). However, significant attenuation of antihypertensive effects was observed at 4 h in the concomitant enalapril and FMP group, compared with the enalapril monotherapy group (p < 0.05; Fig. 1a). The antihypertensive effects of enalapril were not reduced in the concomitant enalapril and FMP (VPP, IPP: 1.75 mg/kg) group (Fig. 1b), which showed significant blood pressure reduction from 1 to 6 h (p < 0.01 and p < 0.05) post administration. Concomitant administration of enalapril and FPP (LKPNM, 10 mg/kg) also produced significant antihypertensive effects 4 and 6 h after administration. However, these were significantly attenuated at 1 and 2 h, as compared with the enalapril monotherapy group (p < 0.05; Fig. 1c). This study indicated that co-administration of FMP or FPP reduced the antihypertensive effects of enalapril. Therefore, these results suggested possible interactions between enalapril and ACE inhibitory peptides from two FOSHU preparations. Because enalapril and FMP/FPP both inhibit ACE activity, they would be expected to have an additive antihypertensive effect. The mechanism underlying the interaction observed in this study may therefore relate to competition for absorption processes in the gut, resulting in reduced enalapril-mediated antihypertensive activity. A previous study has reported that the antihypertensive effects of captopril decreased when this ACE inhibitor was administered concomitantly with the Val-Tyr dipeptide, because of their competition for peptide transporter 1 during intestinal absorption [11]. Similarly, the intestinal absorption of enalapril has been reported to involve peptide transporter 1 [12, 13].

Meanwhile, intestinal absorption of the highly hydrophobic VPP seems to occur via a paracellular pathway involving tight junctions, rather than involving a peptide transporter [14]. Furthermore, the FMP effects disappeared when a lower concentration was employed, perhaps indicating a dose-dependent effect or involvement of a saturable transporter. In addition, intestinal peptide transporters (PEPT1 and PEPT2) exhibit high affinity for endogenous peptides but it was not the case occasionally. It may be probable that VPP exhibits very low affinity to be saturated in such concentration range. Therefore, further investigation is required to evaluate FMP pharmacokinetics and to elucidate the mechanism of the observed interaction with enalapril.

### Long-term administration study

**Fig. 2:**
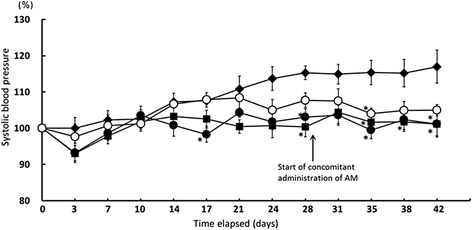
Systolic blood pressure changes in SHR during 6-week oral administration. Systolic blood pressure changes in SHR during 6-week oral administration of enalapril monotherapy (■), concomitant enalapril and FMP (○), or initial enalapril monotherapy supplemented by FMP from day 29 onwards (●). The control group (♦) received distilled water. Values are presented as the mean ± SE (*n* = 5). *p < 0.05 vs control group at the same time point (one-way analysis of variance, followed by Bonferroni/Dunn or Scheffe multiple comparison tests)

The present study found no significant differences in the antihypertensive effects of a single oral administration of enalapril monotherapy and oral enalapril followed by FMP 1 h later (data not shown). For this reason, we maintained a 1-h interval during dosing in the long-term study. As the result, compared to the control group, the enalapril monotherapy group showed significant antihypertensive effects on days 28, 35, 38, and 42 (p < 0.05 for all days, Fig. 2). Although administration of enalapril and FMP with an interval of 1 h between oral doses resulted in significant antihypertensive effects (p < 0.05 on day 35 for the comparison with control animals), a delayed onset was observed in comparison to that observed with enalapril monotherapy. Despite selecting this administration method, it became clear that long-term administration of enalapril and FMP produced a lower antihypertensive effect than did enalapril monotherapy. Although the mechanism underlying this effect is unknown, one possible explanation is an interaction at ACE catalytic sites *in vivo*. Although the precise mechanisms responsible for the *in vivo* antihypertensive effects of FMP, or the IPP/VPP tripeptides, have not been elucidated clearly, they have been suggested to involve ACE inhibition [5], or vasodilator production [15]. IPP and VPP may also target the aorta, where they interact with ACE catalytic sites, inhibiting ACE activity [16–18].

When enalapril monotherapy was supplemented by FMP, significant antihypertensive effects were observed on days 35 and 42 (both p < 0.05) in the delayed combination group. Moreover, these effects were not significantly different from those observed during enalapril monotherapy (Fig. 2). This suggested that FMP administration had no effect on ongoing enalapril treatment. This may relate to the finding by many studies that FMP (or VPP/IPP) only exert their effects in subjects with clinically established hypertension [7, 19–23].

Many previous reports have shown that the long-term intake of FMP, or IPP and VPP tripeptides, effectively lowers blood pressure in SHR [10, 24] and humans [7, 8, 19–22, 25–27]. However, this is the first report of a potential interaction between an ACE inhibitor and a FOSHU product containing ACE inhibitory peptides in SHR with long-term administration.

## Conclusions

The present findings suggested that long-term concomitant intake of FMP and enalapril could influence the antihypertensive effects of this drug. Therefore, they may be beneficial to people who have health concerns about taking ACE inhibitors over extended periods of time.
